# EEG-based motor network biomarkers for identifying target patients with stroke for upper limb rehabilitation and its construct validity

**DOI:** 10.1371/journal.pone.0178822

**Published:** 2017-06-14

**Authors:** Chun-Chuan Chen, Si-Huei Lee, Wei-Jen Wang, Yu-Chen Lin, Mu-Chun Su

**Affiliations:** 1 Department of Biomedical Sciences and Engineering, National Central University, Taoyuan, Taiwan, R. O. C; 2 Department of Physical medicine and Rehabilitation, Taipei Veterans General Hospital, Taiepi, Taiwan, R. O. C; 3 Department of Medicine, College of Medicine, National Yang Ming University, Taipei, Taiwan, R. O. C; 4 Department of Computer Science and Information Engineering, National Central University, Taoyuan, Taiwan, R. O. C; University of Electronic Science and Technology of China, CHINA

## Abstract

Rehabilitation is the main therapeutic approach for reducing poststroke functional deficits in the affected upper limb; however, significant between-patient variability in rehabilitation efficacy indicates the need to target patients who are likely to have clinically significant improvement after treatment. Many studies have determined robust predictors of recovery and treatment gains and yielded many great results using linear approachs. Evidence has emerged that the nonlinearity is a crucial aspect to study the inter-areal communication in human brains and abnormality of oscillatory activities in the motor system is linked to the pathological states. In this study, we hypothesized that combinations of linear and nonlinear (cross-frequency) network connectivity parameters are favourable biomarkers for stratifying patients for upper limb rehabilitation with increased accuracy. We identified the biomarkers by using 37 prerehabilitation electroencephalogram (EEG) datasets during a movement task through effective connectivity and logistic regression analyses. The predictive power of these biomarkers was then tested by using 16 independent datasets (i.e. construct validation). In addition, 14 right handed healthy subjects were also enrolled for comparisons. The result shows that the beta plus gamma or theta network features provided the best classification accuracy of 92%. The predictive value and the sensitivity of these biomarkers were 81.3% and 90.9%, respectively. Subcortical lesion, the time poststroke and initial Wolf Motor Function Test (WMFT) score were identified as the most significant clinical variables affecting the classification accuracy of this predictive model. Moreover, 12 of 14 normal controls were classified as having favourable recovery. In conclusion, EEG-based linear and nonlinear motor network biomarkers are robust and can help clinical decision making.

## Introduction

Motor deficits of the affected upper limb (UL) after stroke affect up to two-thirds of stroke patients and rehabilitation is one of the most frequently used therapeutic approaches for reducing the affected UL impairments. Because the most effective treatment approaches and techniques remain unclear, rehabilitation efficacy significantly varies among patients [1,2], especially in those with moderate initial motor impairment [3,4,5]. Therefore, the ability to identify the target populations who can significantly benefit from treatment is particularly important for maximizing the rehabilitation efficacy [6].

Many studies have determined robust predictors of recovery and treatment gains after stroke, such as age, lesion size and type, time poststroke, and motor function, in addition to neuroimaging findings [7], including cortical activation, network connectivity, and structural integrity [8]. For instance, the motor network connectivity measured with functional magnetic resonance imaging (fMRI) has been shown to facilitate individualized prediction of 86% accuracy [9]. Importantly, cortical oscillations in the five core motor areas, including supplementary motor area (SMA), bilateral primary motor cortex (M1) and premotor cortex (PM) are modulated by hand movements [10,11,12] and by stroke [13,14,15]. After ischemic stroke, the regional θ and γ oscillations were increased while β rhythms were decreased during the acute phase [15], and a frequency-specific parameter based on the (δ +θ)/(α + β) ratio derived from patients' EEG has been used to predict functional outcomes [16]. Recently, Nicolo et al demonstrated that the EEG β coherence between the ipsilesional M1 and all other brain regions was linearly correlated with more favorable motor improvement in patients with subacute stroke [17]. Similarly, Wu et al. identified the linear cortical connectivity in the high β band between ipsilesional M1 and parietal operculum during resting state as biomarkers for predicting motor gains from therapy in chronic stroke patients [18]. Therefore, studies have focused on combinations of multi-modality predictors to increase the predictive value and yielded many good results [4,7,19]. However, these predictors were identified using linear approaches such as coherence and linear regression. Linear methods can extract the most significant features of the data and provide a means of summarizing the system characters, but may not be able to represent all the properties of brain signals accurately [20]. Evidence has emerged that the nonlinearity is a crucial aspect to brain function (for review, see Stam, 2005). Specifially, nonlinear approaches have been widely applied to study the inter-areal communication in human brains in the expression of cross-frequency coupling [21,22,23,24,25,26] and can be supplementary to the linear methods. In addition, the application of most predictors was limited to certain patient populations, for instance, patients in acute-subacute stages and with ischemic stroke. It is still not clear whether these predictors can be generalized to different patient populations. Nevertheless, the construct validity of predictors determined using independent datasets is particularly essential for clinical practice; but, only very few have been properly validated [27].

Considering that nonlinearity is a crucial aspect to study the inter-areal communication and both frequency-specific parameters (evidenced using EEG) and network connectivity (evidenced using EEG and fMRI) are of a prognostic value, we hypothesized that combinations of nonlinear (cross-frequency) and linear (within frequency) network connectivity parameters are favourable biomarkers for stratifying patients for upper limb rehabilitation with increased accuracy. We identified these biomarkers using the pre-rehabilitation EEG data through effective connectivity analysis of dynamic causal modelling (DCM)[28,29] and logistic regression. The predictive power of these biomarkers was then tested by using independent datasets (i.e. construct validation) and the clinical variables were examined to determine significant factors affecting the classification accuracy of these biomarkers.

## Materials and methods

### Ethics statement

This study was approved by the Institutional Review Board of Taipei Veterans General Hospital (approval number: 2010120091A).

### Patients and outcome measurements

Patients with stroke admitted to the Rehabilitation Center at Taipei Veterans General Hospital were included. The inclusion criteria were as follows: (1) first hemiparetic stroke, (2) Brunnstrom stage between stage II and V, (3) no cognitive dysfunction and (4) willingness to participate and sign the informed consent form. The exclusion criteria were as follows: (1) unstable vital signs; (2) irreversible contracture over any of the joints of the upper extremity; (3) a history of surgery, fracture, arthritis, or pain; (4) spasticity (modified Ashworth Scale score of >2); (5) poststroke seizures; (6) heart attack within 3 months; and (7) cortical lesions in the 5 core motor areas. Of the recruited 60 patients, 7 withdrew because of personal reasons. Among the final 53 patients enrolled, EEGs of the first 37 patients were used as training data and those of the remaining 16 patients were used as validation data. This study was approved by the Institutional Review Board of Taipei Veterans General Hospital. All patients provided their written consent. They received 24-hour rehabilitation, 3 times per week for 8 weeks. The UL motor function of all patients was evaluated before and after the intervention by using the Fugl—Meyer Assessment of Physical Performance (FMA) [30], Upper Extremity Performance Evaluation Test for the Elderly (TEMPA) [31], and WMFT [32]. Table 1 lists the patients' demographic and clinical characteristics. Furthermore, we recruited 14 healthy right-handed participants in the control group for comparison.

**Table 1:** Demographic and clinical characteristic of the patients.

dataset	ID	gender	age	time poststroke (month)	hand dominance	stroke type	affected hemisphere	MRI report	Brunnstrom stage (proximal)	Brunnstrom stage(distal)	FMA pre	FMA post	TEMPA pre	TEMPA post	WMFT_pre	WMFT_post	true condition	DCM prediction
T_F	1	M	72	15	R	I	L	brain stem	3	3	23	37	-67	-46	26	37	F	
T_F	2	M	68	12	R	H	L	thalamus intracerebral	4	4	38	50	-48	-33	49	56	F	
T_F	3	M	53	2	R	H	R	basal ganglion ICH	3	2	13	26	-86	-78	31	38	F	
T_F	4	M	59	1	R	I	R	MCA	5	5	59	66	-14	-6	67	73	F	
T_F	5	M	50	5	R	H	L	basal ganglion ICH	3	3	11	19	-72	-64	12	14	F	
T_F	6	M	65	8	R	I	L	post part of MCA	5	4	42	43	-44	-27	55	54	F	
T_F	7	M	59	12	R	I	L	MCA	5	4	35	42	-35	-40	46	43	F	
T_F	8	F	33	6	R	I	R	MCA	2	2	9	8	-93	-87	20	29	F	
T_F	9	M	69	3	R	H	L	thalamus	5	6	42	53	-29	-20	64	66	F	
T_F	10	F	60	1	R	I	R	MCA	2	2	13	26	-93	-84	31	41	F	
T_F	11	M	62	13	R	I	L	basalganglia and thalamus	3	2	15	17	-94	-76	35	36	F	
T_F	12	M	65	5	R	H	L	ICH, pons and midbrain	5	5	52	61	-76	-49	39	47	F	
T_F	13	F	75	4	R	I	R	MCA	4	5	36	52	-57	-29	43	57	F	
T_F	14	M	27	2	L	H	L	Fronto- temporal regions	3	2	17	21	-69	-79	12	23	F	
T_F	15	F	68	2	R	H	R	ICH thalamus	4	5	31	40	-41	-35	54	61	F	
T_F	16	M	63	2	R	I	L	MCA	2	1	8	9	-75	-86	21	36	F	
T_F	17	M	57	2	R	H	L	putamen	4	4	24	35	-64	-50	42	54	F	
T_F	18	M	69	1	R	H	L	thalamus	5	6	43	52	-33	-17	56	60	F	
T_F	19	M	51	1	R	I	L	corona rediata	4	2	22	31	-68	-54	42	49	F	
T_P	20	M	39	15	R	H	L	ICH	3	3	22	26	-70	-68	38	38	P	
T_P	21	M	60	17	R	H	L	ICH putamen	3	2	21	19	-79	-74	35	36	P	
T_P	22	F	38	5	R	H	L	ICH thalamus	4	4	45	49	-35	-25	57	59	P	
T_P	23	M	58	4	R	I	R	MCA	5	5	35	40	-45	-36	50	55	P	
T_P	24	F	44	10	L	I	L	MCA	5	4	36	38	-51	-46	51	53	P	
T_P	25	M	64	12	R	H	R	thalamic and basal ganglia	3	3	17	16	-93	-86	35	32	P	
T_P	26	F	61	3	R	I	R	Fron- topartieal	4	4	36	42	-47	-39	48	52	P	
T_P	27	F	79	1	R	I	R	paramedian area of the pons	5	5	43	44	-26	-27	65	59	P	
T_P	28	M	46	10	R	I	L	medulla	5	5	43	44	-23	-28	62	65	P	
T_P	29	M	50	9	R	I	R	MCA	5	4	51	52	-22	-21	67	64	P	
T_P	30	M	28	9	L	H	L	Fronto- temporal regions	3	3	23	24	-82	-68	42	45	P	
T_P	31	M	57	5	R	H	R	thalamus and brain stem ICH	4	5	34	38	-65	-66	21	29	P	
T_P	32	F	75	5	R	H	R	ICH thalamus	4	5	33	31	-50	-49	42	46	P	
T_P	33	M	54	4	R	I	L	ACA	5	5	55	57	-7	-7	68	68	P	
T_P	34	M	76	5	R	I	L	Psterior corona radiata	3	5	31	25	-49	-43	50	57	P	
T_P	35	F	49	20	R	I	R	middle and superior frontal lobe	5	4	35	37	-51	-55	46	49	P	
T_P	36	M	58	12	R	H	R	thalamus, corona vadiata, lentinucleus	2	1	10	10	-57	-69	12	21	P	
T_P	37	M	45	2	R	I	L	medulla	5	5	42	44	-25	-26	63	65	P	
V	1	F	59	7	R	H	R	MCA	2	4	23	25	-88	-88	36	49.5	F	P
V	2	M	58	8	R	I	L	basalganglia and thalamus	3	3	24	33	-105	-87	46	54	F	F
V	3	M	44	21	R	I	R	basal ganglia	2	2	14	16	-104	-87	43	28	F	F
V	4	M	34	8	R	I	L	ganglion	5	4	38	40	-81	-82	49	60	F	F
V	5	M	46	8	R	H	R	basal ganglion	3	3	18	29	-86	-93	50	59	F	F
V	6	M	44	13	R	I	L	paramedian pontine	4	5	50	56	-28	-7	68	71	F	F
V	7	M	60	1	R	I	R	posterior limb of internal capsule	5	5	52	59	-15	-5	73	71	F	F
V	8	M	39	7	R	I	L	medial medulla and cerebellum	5	4	38	39	-16	-14	72	83	F	F
V	9	M	60	6	R	I	L	caudate nucleus	3	4	30	32	-75	-57	50	51	F	F
V	10	M	53	4	R	H	R	basal ganglion	4	4	38	52	-63	-25	65	68	F	F
V	11	M	51	12	R	H	L	MCA	3	4	22	29	-82	-79	40	40	F	F
V	12	F	27	7	R	I	R	basal ganglion	4	5	33	34	-68	-65	46	45	P	P
V	13	M	64	16	R	I	R	MCA	3	3	21	22	-63	-63	37	37	P	F
V	14	M	55	5	R	I	R	mid brain to pons	3	3	48	51	-60	-52	59	63	P	P
V	15	M	62	10	R	I	L	MCA	4	4	46	47	-42	-68	74	74	P	P
V	16	M	51	14	R	H	R	AVM; fronto-tempro-parietal	3	4	37	43	-44	-48	61	62	P	F

T_F: Training_ Favorable; T_P: Training_ Poor; V: Validity; I: ischemic; H: hemorrhagic; F: favorable; P: poor

### Movement task and EEG preprocessing

Before rehabilitation, the patients were instructed to use their affected hand to perform either shoulder or elbow flexion and extension, depending on their movement ability. The healthy controls performed the right shoulder flexion and extension. All participants sat upright with 0 degree of shoulder flexion. For elbow movements, the affected upper arm rested on an armrest adjusted for the subject’s height. Auditory cues were used to pace the movement (intertrial interval = 10 ± 2 s to avoid anticipation). Fail in completing a movement (angle of movement < 45 degree) was recorded digitally by the therapist pressing a button and this trial was discarded from further analysis. For every 10 success trials, participants took a short break of 1–10 minutes to prevent the distortion of performance and thus results engendered by muscle fatigue. We acquired 80 trials for each participant. All participants were instructed not to blink in the first 2 seconds of each movement.

During hand movements, 32-channel EEG data (10–20 system montage) were measured (sampling rate = 2000 Hz) and preprocessed offline by using SPM8 (Wellcome Trust Centre for Neuroimaging, http://www.fil.ion.ucl.ac.uk/spm/). The EEG data were first epoched with a peristimulus window of −1500 to 2000 milliseconds (time zero indicated the onset of an auditory cue) and filtered from 4 to 48 Hz. Artefact-contaminated trials such as electrooculography or movements (EEG amplitude > 500 μV) were excluded from further analysis. These artefact-free trials were then entered into DCM.

### DCM specification and Bayesian model selection

We adopted 5 sources of the SMA ([-2–2 62]), bilateral M1 ([-41–26 56; 49–27 56]) and PM ([-40–12 52; 40–2 62]) from our previous study [12] as the initial guess. The locations of the 5 predefined areas were optimized individually through the DCM inversion. The model space of the connection architecture was constructed under the following hypotheses. First, intrinsic connections become more nonlinear after stroke, in contrast to being more linear under a healthy state. Second, reduced lateralized activation after stroke results in a less lateralized network [33]. This resulted in 6 models for comparison (see S1 Fig), which have been tested in healthy individuals [12]. Furthermore, we also constructed an all-linear model for comparison. All connections have been structurally identified in the macaque brains [34,35] and functionally tested using fMRI and EEG data in the human brain during motor tasks [36] [37,38]. For given DCM and EEG data, artefact-free epochs were projected from the channel space to sources by using the generalized inverse of a lead field matrix for our chosen sources and then transformed using a time-frequency Morlet wavelet transform (wavelet number 7) over the peristimulus time of -500 to 800 milliseconds. The absolute values of the resulting time—frequency responses were then averaged over artefact-free trials and baseline (−850 to −800 ms) corrected to produce the power spectrum at sources. Bayesian inversion was employed to estimate DCM parameters, particularly the matrices *A* that contain coupling parameters of modulating spectral activity induced by other sources and exogenous (e.g., stimulus) inputs. The details of this standard procedure have been reported in previous studies [12,28,37,38].

To perform a second-level analysis of the model space, the models of patients with left-hand weakness (right hemispheric lesion) were flipped along the mid-sagittal line. Bayesian model selection (BMS) was used to identify the best model under the fixed and random effects assumption at the individual and group levels [39,40,41] for the control and training groups.

### DCM feature extraction

**Fig 1:**
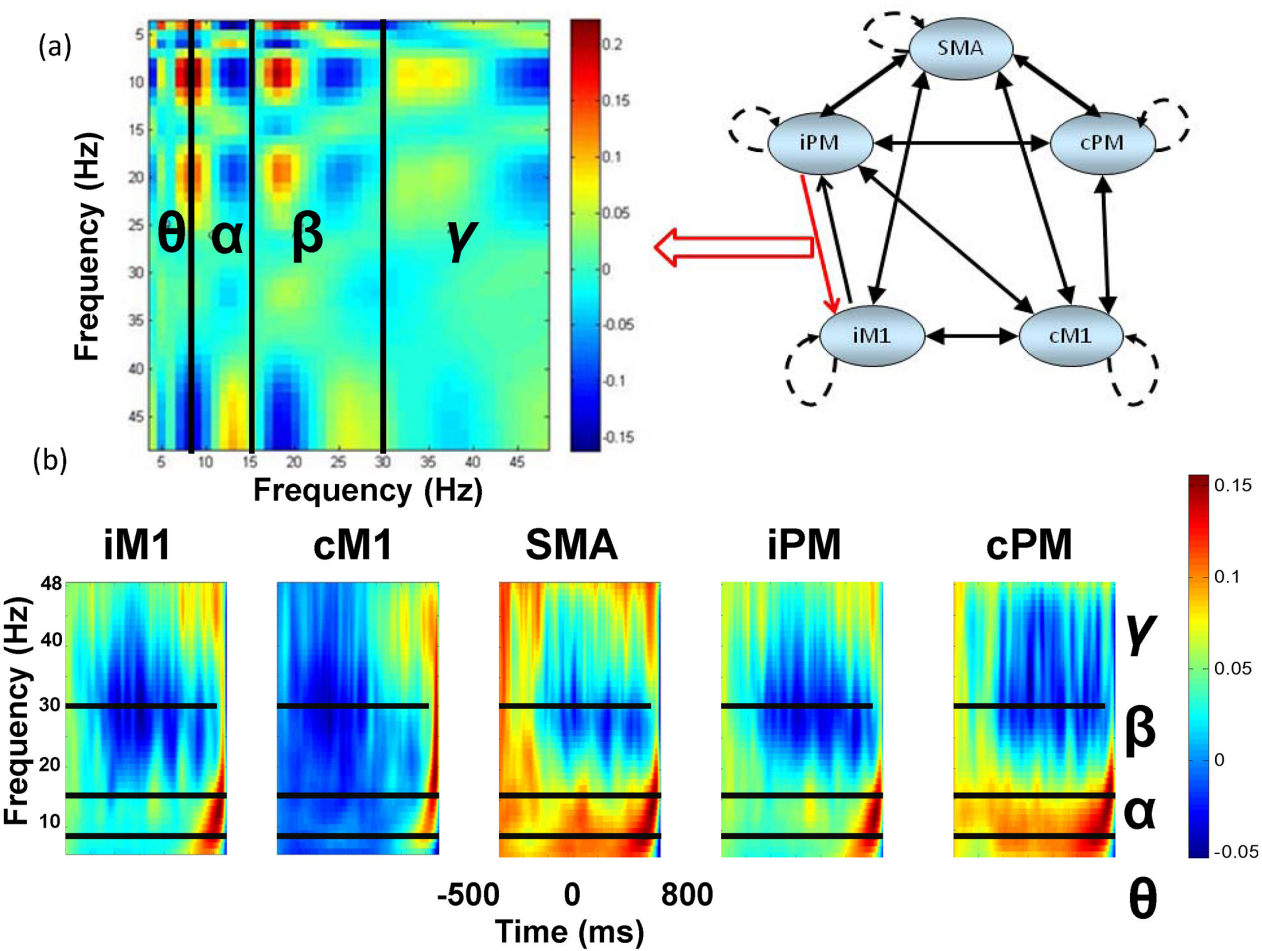
Illustrations of frequency- and connection-specific parameters (a) in 5 core motor cortices and the frequency-specific spectrum dynamics (b) at the five core motor cortices.

After establishing the best model, we divided the coupling parameters in the core motor cortical network into 4 frequency bands, namely ɵ (4–8 Hz), α (8–15 Hz), β (15–30 Hz), and γ (30–48 Hz), and 2 mechanisms, namely excitatory (positive) and inhibitory (negative); we averaged them across frequencies to give 8 parameters for each connection (Fig 1a). After BMS, we can then determine the number of attributes for each subject that entered into the classifier for constructing the predictive model. For example, there were 18 connections in the model profiled in Fig 1a, including 9 reciprocal connections between SMA-cM1, SMA-iM1, SMA-iPM, SMA-cPM, iPM-cPM, iPM-iM1, cPM-cM1, iPM-cM1 and iM1-cM1 and this yielded 144 attributes (8 parameters x 18 connections). For comparison, spectra over the 5 core areas were also averaged over the prespecified 4 frequency bands and mechanisms (positive and negative) to give 8 parameters for each source (Fig 1b). Hence, 40 attributes were obtained for each participant.

### Classification and construct validity

The first 37 patients recruited (i.e., the training data set) were categorized as either having favorable (n = 19) or poor recovery (n = 18) according to whether their postrehabilitation improvement in FMA, WMFT, or TEMPA reached the 10% level of the maximum score [42] (Table 1). The DCM features of the best model at the group level were used in a logistic regression for two-class classification (i.e., favorable and poor). Five-fold cross-validation was used to evaluate the classification accuracy of different combinations of frequency-specific features derived from DCM. After identifying the optimal combination of frequency-specific features, we used a backward elimination procedure to select significant connection-specific features [43,44].

Sixteen new patient datasets were used to validate the predictive model at the individual level. For comparison, we performed a dichotomous classification on the clinical factors of the validation dataset by using cutoffs that best separated the favorable from the poor outcomes. Finally, 14 datasets of healthy participants were tested through the predictive model to determine the degree to which the model reflects the normal motor network. The program used for classification and the identified predictive model in this study can be downloaded from http://140.115.52.12/.

### Statistical tests

A 2-sample t-test was used to examine whether the clinical characteristic of patients significantly differed between datasets. Furthermore, after establishing the best predictive model, we statistically analyzed the frequency- and connection-specific parameters in the model by using analysis of variance to determine whether these predictors were significantly consistent across the patients. Statistical significance was set at *P* < .05.

## Results

### Demographic characteristics at the baseline and improvement after rehabilitation

Table 1 listed the demographic characteristics of all patients at the baseline and improvement after rehabilitation and Table 2 summarized the statistic results of demographic and clinical characteristic. The statistical result confirmed that the validation dataset resembled the training dataset, suggesting a similarity among recruited patients. We observed no significant between-dataset differences in age, time poststroke, or initial impairment measured using the Brunnstrom stage, FMA, and TEMPA (Table 2). However, the WMFT scores were significantly lower in the training dataset than in the validation dataset (*P* = .04). Notably, age (*P* = .07) and time poststroke (*P* = .11) tended to differ between the 2 datasets, but the difference was not significant. The participants were significantly younger (*P* = .02) and the WMFT scores were significantly higher (*P* = .048) in the validation dataset than in the training dataset. Regarding the clinical characteristics of the patients in the training dataset, only time poststroke was significantly shorter in the favorable outcome subgroup than in the poor outcome subgroup (*P* = .03).

**Table 2:** The statistic results of demographic and clinical characteristic.

Dataset	Training dataset (n = 37)			Validity dataset (n = 16)		Between datasets	Between Favorable subsets
Outcome	n = 19	Poor n = 18	P value	Favorable n = 11	Poor n = 5	P value	P value
Gender (F/M)	4/15	6/12		1/10	1/4		
Affected hemisphere (R/L)	6/13	9/9		5/6	1/4		
Lesion site (cortical/ subcortical)	8/11	6/12		1/10	3/2		
Stroke type (ischemic/ hemorrhagic)	10/9	10/8		7/4	4/1		
Hand dominance (R/L)	18/1	16/2		11/0	5/0		
Age (mean±SD)	59.21±12.40	54.50±13.75	0.14	49.81±9.05	48.19±14.55	0.07	0.02*
Time poststroke (month) (mean±SD)	5.10±4.63	8.22±5.39	0.03*	8.64±5.26	10.83±6.18	0.11	0.08
Brunnstrom’s stage (proximal)	3.79±1.18	4.06±0.99	0.46	3.54±1.13	3.20±0.48	0.12	0.58
Brunnstrom’s stage (distal)	3.52±1.54	4.00±1.18	0.30	3.81±0.87	3.33±1.63	0.86	0.51
Pre-FMA scales (mean±SD)	28.05±22.97	34.61±11.87	0.19	29.04±14.98	32.58±12.23	0.53	0.50
FMA improvement (mean±SD)	8.10± 4.85	1.33± 2.89	<0.0001*	5.63±4.31	2.4±2.19	0.88	0.16
	4.81±5.24			4.62±4.92			
Pre TEMPA (mean±SD)	-60.95±21.49	-48.72±23.24	0.12	-67.55±33.07	-48.29±11.70	0.29	0.57
TEMPA improvement (mean±SD)	10.42±10.66	2.44±6.24	0.009*	10.81±13.02	4.39±3.96	0.94	0.932
	6.54±9.57			6.25±9.40			
Pre WMFT (mean±SD)	39.21±15.92	47.33±15.53	0.13	52.00±16.00	45.52±21.59	0.04*	0.048*
WMFT improvement (mean±SD)	6.789+4.82	2.27+3.73	0.003*	3.86+8.08	1.99+2.75	0.38	0.29
	4.59 ±4.95			2.90 ±5.07			

*: P<0.05

Regarding the treatment effect of rehabilitation, the mean improvement scores in the training and validation datasets were 4.81 and 4.62 for the FMA, 6.54 and 6.25 for the TEMPA, and 4.59 and 2.90 for the WMFT, respectively. The mean improvement scores did not significantly differ between the 2 datasets (*P* > .05). However, in the training data set, all improvement scores were higher in the favorable outcome subgroup than in the poor outcome subgroup, indicating that the patients with favorable outcomes were adequately separated from those with poor outcomes. In addition, improvement scores did not significantly differ between the 2 favorable outcome subsets.

### BMS result

**Fig 2:**
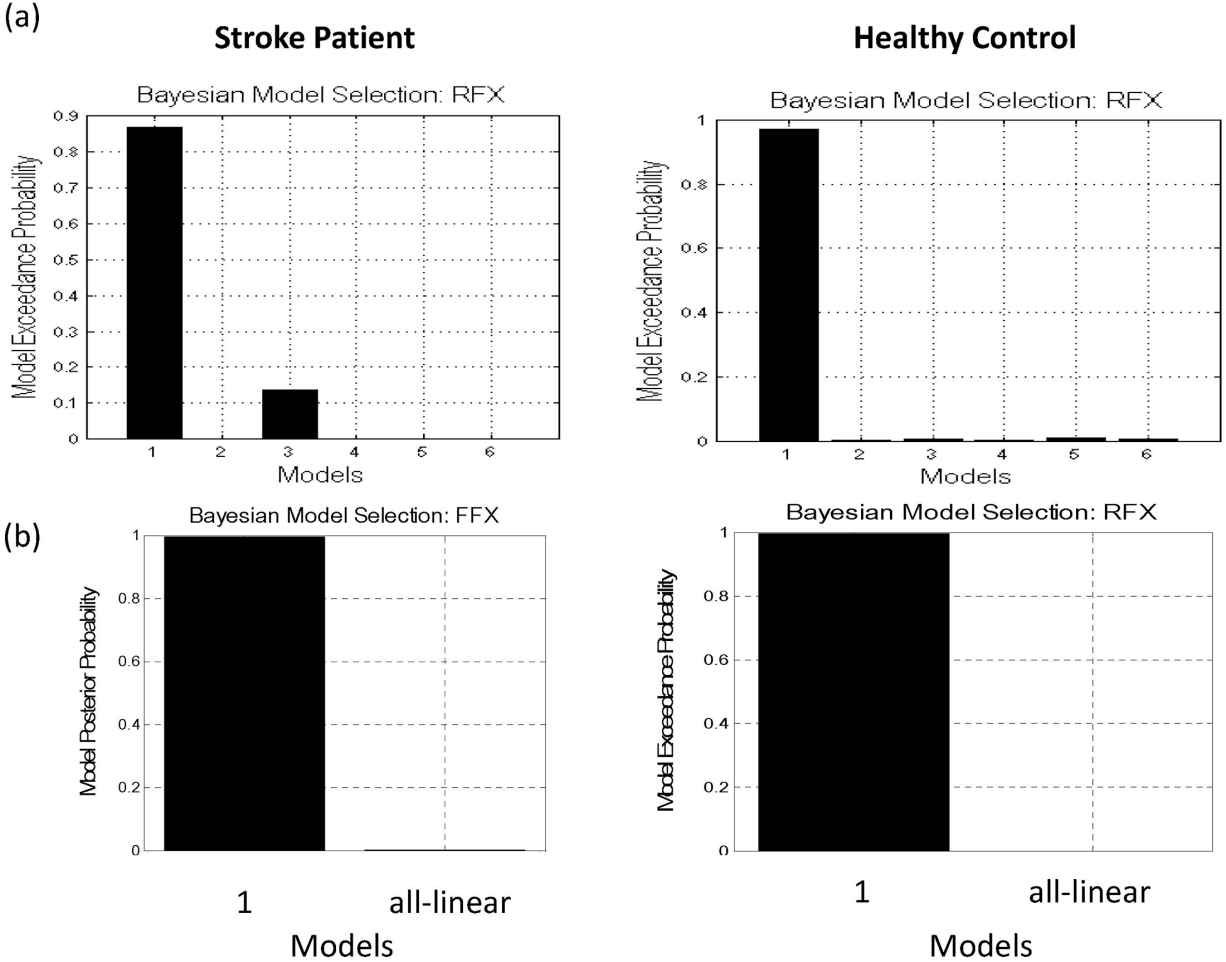
(a) BMS results at the group level under RFX for the patients (left) and the controls (right) both confirmed that Model 1 was the most likely model with exceedance probabilities of 0.87 and 0.97, respectively. (b) Model comparison of nonlinear+linear (Model 1) and all-linear model under FFX (left) and RFX (right).

The BMS results revealed that DCM1 and DCM3 were the best models for 19 and 18 patients in the training data set, respectively, at the individual level. At the group level, the BMS results under random effects assumption identified DCM1 as the optimal model for the training group, with an exceedance probability of 0.87 (Fig 2a, upper panel). Regarding the control group, under RFX, DCM1 was identified as the winning model for all at the individual level and at the group level with an exceedance probability of 0.97 (Fig 2a, lower panel). These results indicated that among the models being tested, DCM1 (shown in Fig 1a, right) was the most optimal for both the patients and healthy controls. We then further compared DCM1 with the all-linear model at both the individual and group level. The BMS results of the training dataset showed an overwhelming significance of DCM1 over all-linear model under FFX and RFX assumptions (Fig 2b).

### Frequency-specific features and classification accuracy

**Fig 3:**
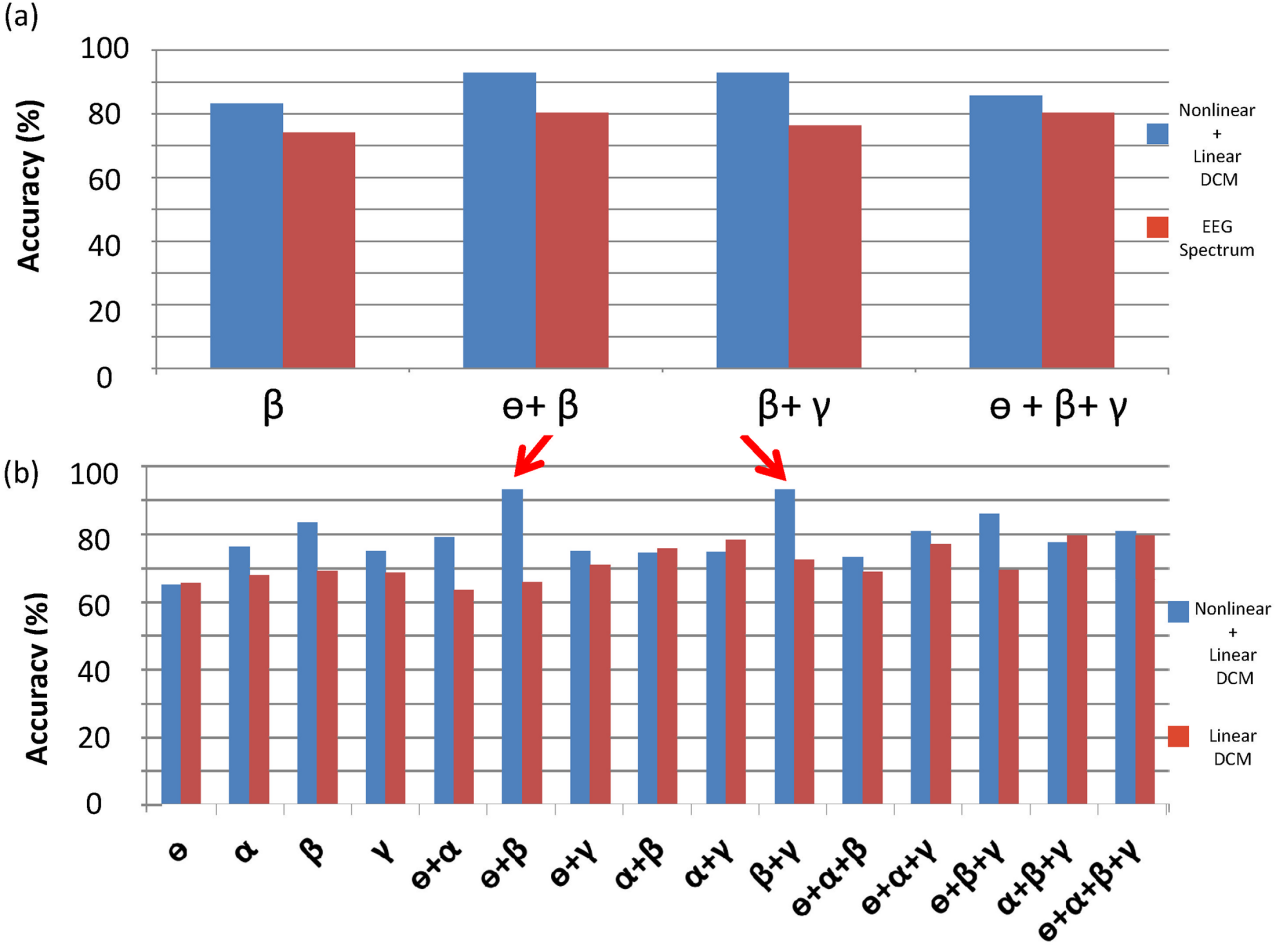
(a) Comparison of classification accuracy between DCM features (blue bars) and source spectral features (red bars). (b) Impact of frequency-dependent linear (red bard) and nonlinear (+linear) network DCM (blue bars) features on classification accuracy. The red arrows indicate the best accuracy.

We compared the classification accuracy of frequency-specific features derived from DCM1 with that of features derived from the spectrum in the 5 motor areas. The features of the motor network (i.e. DCM features) provided higher classification accuracy (up to 90%) than did the frequency-related source power alone (up to 80%; Fig 3a). Furthermore, we investigated the effect of the frequency and nonlinearity on the classification accuracy. Fig 3b presents a plot of all the combinations of frequency-specific features against the classification accuracy. We observed that, under DCM1, β oscillation was the most crucial component in the motor network, yielding an 83.1% ± 3.9% accuracy, followed by alpha and gamma rhythms. The β plus γ and β plus ɵ features yielded the highest accuracies (92.9% ± 5.0% and 92.9% ± 4.8%, respectively; Fig 3b, red arrows). However, both the combination of β and α features and the use of all features did not improve the classification accuracy. Regarding to the linear features derived from the all-linear DCM, we found the best accuracy was approximately 80% when using α+β+γ features.

### Network –specific features

**Fig 4:**
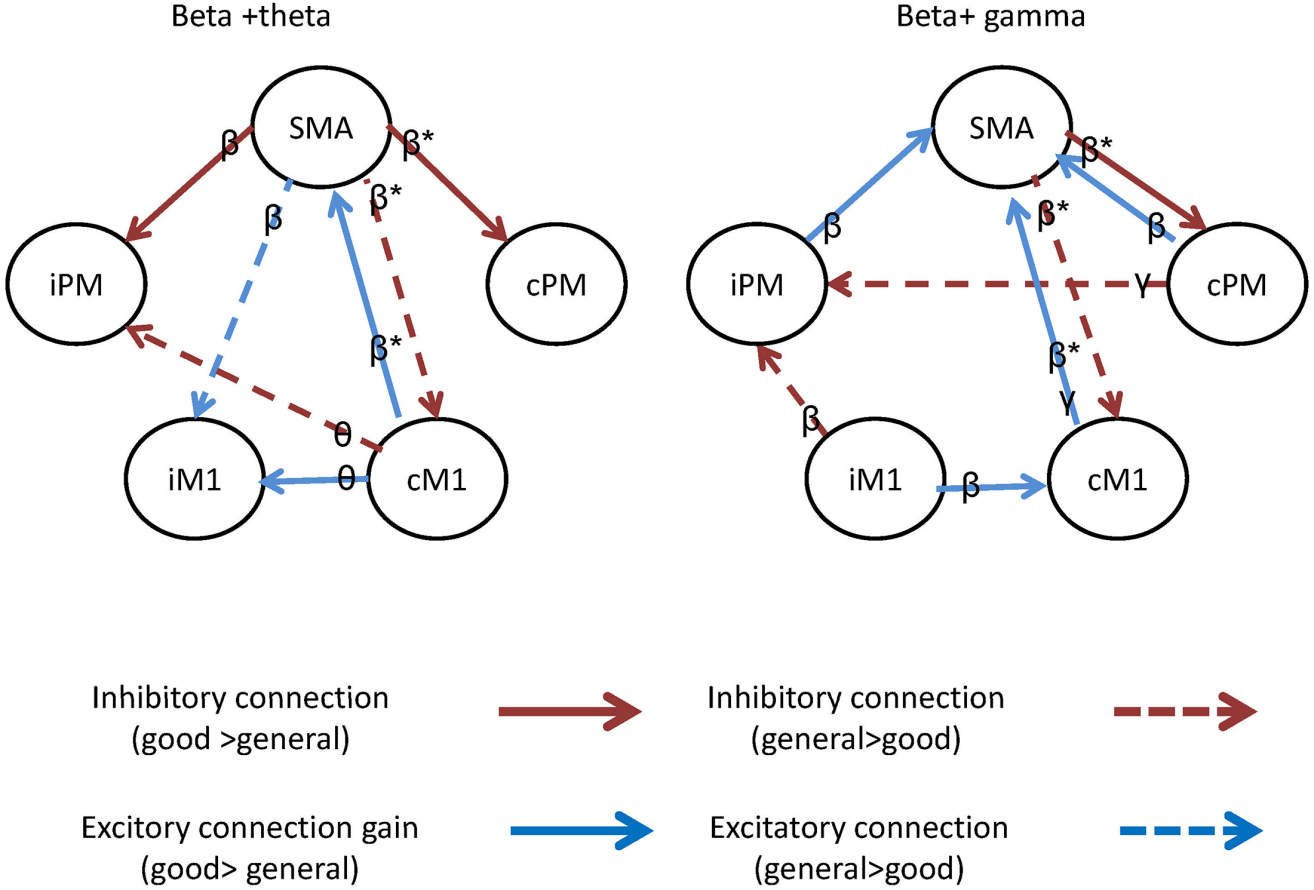
(a)Significant network- and frequency-specific biomarkers of beta plus theta (left) and beta plus gamma (right) rhythms identified by the backward elimination procedure. The asterisks indicate the 3 common connections in both predictive models.

After identifying the optimal combination of frequency-specific features, we used a backward elimination procedure to select significant area-specific features. We determined 7 and 8 connections for β plus ɵ (Fig 4, left) and β plus γ (Fig 4, right) features, respectively, which could effectively differentiate between the patients with favorable and poor recovery. Three common β connections existed in both network models (marked with asterisks): 2 originating from SMA and 1 from cM1. Specifically, in the favorable recovery group, the SMA β connection exerted a higher negative effect on cPM but a lower inhibitory effect on cM1 while cM1 β activity facilitated SMA rhythms. In other words, we identified a greater involvement of the SMA and cM1, but low engagement of cPM, to be critical for treatment gains. However, none of the network-specific features identified reached significance in the ANOVA test (*P* > .05) at the group level to differentiate between the patients with favorable and poor recovery.

### Construct validity and clinical variables with predictive value

We validated our model by using 16 new datasets of the patients with stroke. The overall accuracy, sensitivity, and positive predictive value were 81.3%, 90.9%, and 83.3%, respectively (Table 3). Furthermore, we identified that subcortical lesions, time poststroke, and initial impairment scores measured using the WMFT were the most significant clinical variables affecting the classification accuracy in this model (Table 4). When the motor cortices were intact (i.e., subcortical lesions) and time poststroke was within 6 months, the classification rate was accurate (100% accuracy). The classification accuracy decreased with an increase in time poststroke, and it further decreased considerably when the time poststroke was >12 months (50% accuracy). In addition, the outcome was predictable in this model when the initial WMFT scores were above 45 (91% accuracy). Although age significantly differed between the training and validation datasets, it did not strongly affect the classification. The classification accuracies for the patients aged older than and younger than 55 years were 71% and 88%, respectively (Table 4). Furthermore, the predictive value of the network biomarkers was always superior to the prediction accuracy obtained by the dichotomous classification. Of the 14 control participants used to test the predictive model, 12 were predicted to have favorable outcomes and 2 to have poor outcomes.

**Table 3:** The sensitivity and the positive predictive value of the neuromarkers.

		Outcome		
		Favorable	Poor	
Predicted condition	Favorable	10	2	Positive predictive value = 10/12 = 83.3%
	Poor	1	3	
		Sensitivity = 10/11 = 90.9%	Specificity = 3/5 = 60%	Accuracy = 13/16 = 81.3%

**Table 4:** Clinical variables affecting the prediction.

Clinical variables	EEG Prediction accuracy (*n*)			Best dichotomic classification accuracy (n)	
Lesion area	Subcortical (*n* = 12)	100% (n = 12)			
	Cortical (*n* = 4)	25% (n = 1)			
Time poststroke (months)	Acute-subacute (1~6) (*n* = 5)	100% (n = 5)	91% (n = 11)		
	Chronic (7~12) (*n* = 7)	85% (n = 6)			
	Chronic (>12) (*n* = 4)	50% (n = 2)			
	< = 9 month (n = 10)	90% (n = 9)		< = 9 month (n = 10)	80% (n = 8)
	> 9 month (n = 6)	66% (n = 4)		> 9 month (n = 6)	50% (n = 3)
Initial WMFT score	< = 45 (*n* = 4)	50% (n = 2)			
	>45 (*n* = 12)	91% (n = 11)			
	< = 50 (*n* = 9)	77% (n = 7)		< = 50 (*n* = 9)	77% (n = 7)
	>50 (*n* = 7)	85% (n = 6)		>50 (*n* = 7)	42% (n = 3)
Age	< = 50 (*n* = 9)	77% (n = 7)		< = 50 (*n* = 9)	77% (n = 7)
	>50 (*n* = 7)	85% (n = 6)		>50 (*n* = 7)	42% (n = 3)
	<55 (*n* = 9)	88% (n = 8)			
	> = 55(*n* = 7)	71% (n = 5)			

## Discussion

### Prior requirements for the application of the predictive model

In this study, we identified the linear and nonlinear motor network biomarkers using the prerehabilitation EEG data of a heterogeneous stroke population. By examining the clinical variables affecting the classification accuracy using independent data sets, we further recognized the patient populations for the predictive model.

### Effect of time poststroke

The time poststroke in the training data set was significantly shorter in the patients with favorable outcomes (5.10 ± 4.63 mo) than in those with poor outcomes (8.22 ± 5.39 mo; *P* = .03), and this result supports the sensitive period for recovery (≤6 mo). In other words, stroke patients should receive the rehabilitation treatment as early as possible to enhance the efficacy. However, this result does not indicate that the predictive model can be applied only to patients close to their chronic stage, because the favorable group used in the construction of the predictive model had an average time poststroke of 5 months. The mean time poststroke was averaged across a broad range of time poststroke (1–15 mo) in this data set and the time poststroke of 10 of the 19 patients in the favorable group of the training data set was within 3 months (Table 1). In addition, because of the significant between-participant variability in neuroplasticity in response to rehabilitation, the sensitive period of “<6 months” is not a highly inferential factor. In the training data set, 9 of the 22 patients in the early-to-late subacute phase (≤6 mo) did not exhibit significant improvement after rehabilitation, whereas 5 of the 15 patients in the chronic phase demonstrated significant improvement (Table 1). Moreover, the factor of a time poststroke within 9 months became the best cutoff for the dichotomous classification. Therefore, by using an independent data set for validity testing, we demonstrate that patients having a time poststroke within 12 months are suitable for this predictive model (91% overall accuracy), with the model exhibiting excellent performance for patients with a time poststroke within 6 months (100% accuracy; Table 4). Notably, the performance of this predictive model was superior to the best accuracy of the dichotomous classification.

On the basis of our result that 85.71% healthy controls were classified as having favorable outcomes, we can infer that the predictive model of the motor network pattern resembles the normal motor network to a certain degree, but not completely. In addition, the classification accuracy decreased with an increase in the time poststroke, suggesting that recovery and/or maladaptation over time occurs through mechanisms that alter network patterns.

### Effect of lesion site and initial WMFT scores

In addition to the time poststroke, we observed that the subcortical lesion and an initial WMFT score of >45 are also prerequisites for the application of this model. In the training data set, 23 of the 37 patients had a lesion at the subcortical level, and the validation data suggested that the best prediction could be obtained for subcortical lesion sites. Because electroencephalography measures the electrophysiological activity of cortical areas, any damage to the cortex would be expected to perturb the patterns and severely degrade the classification accuracy. By contrast, when the motor cortices are intact, the classification is accurate, as observed in this study. This result also indicates that the subcortical lesion consequently caused cortical network alternations, which are crucial in identifying patients who are responding to rehabilitation.

Only the initial WMFT scores significantly differed between the 2 datasets and 2 favorable subsets. The mean WMFT scores were significantly lower in the training data set than in the validation data set; in addition, the mean WMFT scores were significantly lower in the favorable group of the training data set than in that of the validation data set. It seems that a lower initial WMFT score appeared to predict better outcomes, as observed in the training data set. However, a similar trend was not observed in the validation data set because only a few patients (n = 5) were included in the poor outcome subset. Through dichotomous prediction, we observed that a WMFT score of <50 predicted better outcomes; however, the best accuracy was only 77%. By using the EEG predictive model, we identified a WMFT score of >45 to be inferential, with an accuracy of 91%. By contrast, this result did not support that a lower initial WMFT score could predict better outcomes as 8 of the 12 patients having a WMFT score of >45 were accurately predicted to have favorable outcomes by the EEG model.

In summary, patients who have subcortical lesions, an initial WMFT score of >45, and a time poststroke within 12 months are the most suitable candidates for this predictive model.

### Benefits of favorable outcomes

Measuring rehabilitation outcomes is challenging because of the heterogeneity of stroke. Various measures are currently used in clinical and research practice, but no single measure is universally acceptable or sufficient for recording the minimal clinically important difference [45]. Therefore, in this study, a patient having an improvement of 6.6 on the FMA, 7.5 on the WMFT, or 16.2 on the TEMPA (i.e. 10% increase of the maximum score) was considered to have favorable outcomes. The improvement of 10% is the minimum effect that has been previously considered to be of clinical significance [42] and the FMA and WMFT are highly recommended for measuring the outcomes of patients with stroke [46]. The reported minimal clinically important difference values for chronic patients were 6 for the lower-extremity FMA [47] and 1–1.2 for the WMFT functional score [48]. Therefore, our definition of improvement is beyond the result of chance or increasing familiarity with the test observed in chronic patients. Nevertheless, the statistical results suggest that patients were adequately separated and the classification results are good, thus indicating that this definition is sufficient for our goal.

### Functional significance of frequency- and network-specific biomarkers

In this study, we observed that the DCM derived network features can lead to the best prediction accuracy, thereby confirmed our hypothesis of combining linear and nonlinear (cross-frequency) network parameters to be favourable biomarkers. Furthermore, our result also suggested that the network parameters can add some predictive values to the accuracy as the frequency-specific network features outperformed the frequency-specific source power spectrum by 10%. Nevertheless, the frequency-specific source power spectrum along can provide up to 80% prediction accuracy, indicating the informative value for clinical management and prognostication of outcomes [14,49]. In addition, two possible predictive models (β+γ and β+θ) yielded relatively equal classification accuracy levels and we identified 3 consistent β connections in both network models. All the 3 connections located in the contralesional hemisphere and were connected to the SMA in the β band, highlighting the importance of the intact hemisphere and SMA in recovery. Specifically, our findings of higher SMA activity and lower inhibitory connection between the SMA and cM1 in the favorable recovery group are in agreement with previous studies that have reported that increased activity in the SMA may play a role in recovery [50] and that enhanced β connectivity between the SMA and cM1 is believed to subserve the control of the recovered hand [13]. Moreover, many studies using different methods and devices have confirmed a significant involvement of cM1 as compensatory mechanisms to recovery[51] [52,53,54]. Our finding, that the inhibition of cM1 activity was higher in the patients with poor recovery, may be one of the reasons responsible for poor recovery. Furthermore, a study reported that greater recruitment of cPM helps patients with more severe impairment to control the affected hand [55]. Our findings can explain the underlying mechanisms for these observations—the increase in cPM activity in patients with more severe impairment may be due to the insufficient inhibition from the SMA after stroke that altered the brain network. In summary, SMA β oscillations are the most prominent biomarkers for identifying target patients with stroke for rehabilitation. Regarding the translational values of these biomarkers, SMA activities could be the modulation target for rTMS.

### Importance of nonlinear coupling

Nonlinear coupling in EEG/MEG signals has been seen in a variety of tasks, systems and pathological conditions, for instance, during face processing [56] or as an index of the depth of anaesthesia [57,58], though the nonlinearity may differed in the expressions of cross-frequency coupling. In the motor system, it has been shown that coupling between regions is nonlinear during the performance of a simple motor task [12,37,38]. This nonlinearity was thought to reflect the mechanisms at the level of post-synaptic receptors (e.g., NMDA receptors) that afferent inputs from other regions exert nonlinear effects on intrinsic dynamics [12]. In this study, the result of model comparison strongly points to nonlinear and linear connections (i.e. DCM1) (Fig 2b) for explaining the observed spectral dynamics after stroke. Furthermore, in support of our hypothesis, these nonlinear coupling features are informative as the prediction accuracy was improved when compared to the all-linear features (Fig 3b).

### Spatial resolution of electroencephalography

Unprocessed scalp potentials derived from EEG systems involving <64 channels provide only a coarse spatial resolution [59]; therefore, there is a concern that the cortical activities of the M1 and PM may not have been appropriately separated in this study. We compared the mean spectral difference (msd) between the M1 and PM in both hemispheres by using all 37 patients’ EEG data in the training data set. The statistical result suggested significant spectral differences between the iM1 and iPM (msd = −12.21%; *t* = −71.234, *P* < .0001) and between cM1 and cPM (msd = 26.49%;*t* = 154.5147, *P* < .0001). However, the mean differences of −12.21% and 26.49% also indicate a similarity of >70% in power. Because of the lack of a gold standard for comparison, additional studies using high-density EEG or magnetoencephalography may help derive such a standard. Nevertheless, it has been shown that 19-electrode array is adequate to detect the EEG based frequency-specific markers for predicting the clinical outcome after stroke [60].

### Study considerations and limitations

First, all the patients underwent rehabilitation for 24 hours, and we considered that improvements in clinical scores represented the intervention outcome. However, 27 of the 53 patients having a time poststroke within 6 months may still experience spontaneous recovery and contribute to functional improvement; nevertheless, only 17 of the 27 patients in the early-to-late subacute phase but 13 of the 26 patients in the chronic phase exhibited significant improvement (Table 1). The observed improvement cannot be completely explained by spontaneous recovery. This consideration instead reflects that intervention also plays a role in promoting recovery. Second, because we recruited a heterogeneous stroke population to construct the predictive model, the heterogeneity of patients with stroke rendered any in-depth interpretation of the observed neurophysiological difference between the favorable and poor outcome groups difficult. Thus, we discussed only the possible functional roles of 3 common connections in both predictive models, although some other connections in the predictive model may also be informative. Finally, the motor task used here may confound the results. Because the movements of the proximal part of the UL are partly controlled from the contralesional hemisphere [61], the finding that the intact hemisphere may be crucial in recovery is attributable to the uncrossed descending motor pathways. Additional studies using a set of homogeneous patients and a task that entails moving the distal part of the hand can provide insights into the underlying mechanisms.

In conclusion, poststroke linear and nonlinear features in the motor network are favorable biomarkers that can accurately predict treatment gains. The predictive value and the sensitivity of these biomarkers were 81.3% and 90.9%, respectively. Specifically, SMA beta oscillations were identified as the most crucial biomarker in the motor network. Our findings can help physicians in making clinical decisions, such as suggesting an alternative treatment along with rehabilitation at an early stage, and guide the design of rehabilitation strategies, consequently facilitating overall rehabilitation efficacy.

## Supporting information

S1 Fig Architectures of the plausible models. i: ipsilesional; c: contralesional; M1: primary motor cortex; PM: premotor cortex; SMA: supplementary motor area.
(TIF)

## References

[1] Legg L, Drummond A, Leonardi-Bee J, Gladman JR, Corr S, Donkervoort M, et al (2007) Occupational therapy for patients with problems in personal activities of daily living after stroke: systematic review of randomised trials. BMJ 335: 922 doi: 10.1136/bmj.39343.466863.55 17901469 10.1136/bmj.39343.466863.55 PMC2048861

[2] Steultjens EM, Dekker J, Bouter LM, van de Nes JC, Cup EH, van den Ende CH (2003) Occupational therapy for stroke patients: a systematic review. Stroke 34: 676–687. 10.1161/01.STR.0000057576.77308.30 12624291

[3] Prabhakaran S, Zarahn E, Riley C, Speizer A, Chong JY, Lazar RM, et al (2008) Inter-individual variability in the capacity for motor recovery after ischemic stroke. Neurorehabil Neural Repair 22: 64–71. doi: 10.1177/1545968307305302 17687024 10.1177/1545968307305302

[4] Stinear CM, Barber PA, Petoe M, Anwar S, Byblow WD (2012) The PREP algorithm predicts potential for upper limb recovery after stroke. Brain 135: 2527–2535. doi: 10.1093/brain/aws146 22689909 10.1093/brain/aws146

[5] Zarahn E, Alon L, Ryan SL, Lazar RM, Vry MS, Weiller C, et al (2011) Prediction of motor recovery using initial impairment and fMRI 48 h poststroke. Cereb Cortex 21: 2712–2721. doi: 10.1093/cercor/bhr047 21527788 10.1093/cercor/bhr047 PMC3209795

[6] Burke E, Cramer SC (2013) Biomarkers and predictors of restorative therapy effects after stroke. Curr Neurol Neurosci Rep 13: 329 doi: 10.1007/s11910-012-0329-9 23299824 10.1007/s11910-012-0329-9 PMC3580200

[7] Burke Quinlan E, Dodakian L, See J, McKenzie A, Le V, Wojnowicz M, et al (2015) Neural function, injury, and stroke subtype predict treatment gains after stroke. Ann Neurol 77: 132–145. doi: 10.1002/ana.24309 25382315 10.1002/ana.24309 PMC4293339

[8] Stinear C (2010) Prediction of recovery of motor function after stroke. Lancet Neurol 9: 1228–1232. doi: 10.1016/S1474-4422(10)70247-7 21035399 10.1016/S1474-4422(10)70247-7

[9] Rehme AK, Volz LJ, Feis DL, Eickhoff SB, Fink GR, Grefkes C (2015) Individual prediction of chronic motor outcome in the acute post-stroke stage: Behavioral parameters versus functional imaging. Hum Brain Mapp 36: 4553–4565. doi: 10.1002/hbm.22936 26381168 10.1002/hbm.22936 PMC4619153

[10] Kilner JM, Salenius S, Baker SN, Jackson A, Hari R, Lemon RN (2003) Task-dependent modulations of cortical oscillatory activity in human subjects during a bimanual precision grip task. Neuroimage 18: 67–73. 12507444 10.1006/nimg.2002.1322

[11] Omlor W, Patino L, Hepp-Reymond MC, Kristeva R (2007) Gamma-range corticomuscular coherence during dynamic force output. Neuroimage 34: 1191–1198. doi: 10.1016/j.neuroimage.2006.10.018 17182258 10.1016/j.neuroimage.2006.10.018

[12] Chen CC, Kilner JM, Friston KJ, Kiebel SJ, Jolly RK, Ward NS (2010) Nonlinear coupling in the human motor system. J Neurosci 30: 8393–8399. doi: 10.1523/JNEUROSCI.1194-09.2010 20573886 10.1523/JNEUROSCI.1194-09.2010 PMC2923068

[13] Gerloff C, Bushara K, Sailer A, Wassermann EM, Chen R, Matsuoka T, et al (2006) Multimodal imaging of brain reorganization in motor areas of the contralesional hemisphere of well recovered patients after capsular stroke. Brain 129: 791–808. doi: 10.1093/brain/awh713 16364955 10.1093/brain/awh713

[14] Finnigan S, van Putten MJ (2013) EEG in ischaemic stroke: quantitative EEG can uniquely inform (sub-)acute prognoses and clinical management. Clin Neurophysiol 124: 10–19. doi: 10.1016/j.clinph.2012.07.003 22858178 10.1016/j.clinph.2012.07.003

[15] Rabiller G, He JW, Nishijima Y, Wong A, Liu J (2015) Perturbation of Brain Oscillations after Ischemic Stroke: A Potential Biomarker for Post-Stroke Function and Therapy. Int J Mol Sci 16: 25605–25640. doi: 10.3390/ijms161025605 26516838 10.3390/ijms161025605 PMC4632818

[16] Sheorajpanday RV, Nagels G, Weeren AJ, van Putten MJ, De Deyn PP (2011) Quantitative EEG in ischemic stroke: correlation with functional status after 6 months. Clin Neurophysiol 122: 874–883. doi: 10.1016/j.clinph.2010.07.028 20961806 10.1016/j.clinph.2010.07.028

[17] Nicolo P, Rizk S, Magnin C, Pietro MD, Schnider A, Guggisberg AG (2015) Coherent neural oscillations predict future motor and language improvement after stroke. Brain 138: 3048–3060. doi: 10.1093/brain/awv200 26163304 10.1093/brain/awv200

[18] Wu J, Quinlan EB, Dodakian L, McKenzie A, Kathuria N, Zhou RJ, et al (2015) Connectivity measures are robust biomarkers of cortical function and plasticity after stroke. Brain. 10.1093/brain/awv156 PMC4840951 26070983

[19] Price CJ, Seghier ML, Leff AP (2010) Predicting language outcome and recovery after stroke: the PLORAS system. Nat Rev Neurol 6: 202–210. doi: 10.1038/nrneurol.2010.15 20212513 10.1038/nrneurol.2010.15 PMC3556582

[20] Micheloyannis S, Vourkas M, Bizas M, Simos P, Stam CJ (2003) Changes in linear and nonlinear EEG measures as a function of task complexity: evidence for local and distant signal synchronization. Brain Topogr 15: 239–247. 12866828 10.1023/a:1023962125598

[21] Breakspear M (2002) Nonlinear phase desynchronization in human electroencephalographic data. Hum Brain Mapp 15: 175–198. 11835608 10.1002/hbm.10011 PMC6871870

[22] Chen CC, Henson RN, Stephan KE, Kilner JM, Friston KJ (2009) Forward and backward connections in the brain: A DCM study of functional asymmetries. Neuroimage in press. 10.1016/j.neuroimage.2008.12.041 19162203

[23] Jensen O, Colgin LL (2007) Cross-frequency coupling between neuronal oscillations. Trends Cogn Sci 11: 267–269. doi: 10.1016/j.tics.2007.05.003 17548233 10.1016/j.tics.2007.05.003

[24] Tallon-Baudry C, Bertrand O (1999) Oscillatory gamma activity in humans and its role in object representation. Trends Cogn Sci 3: 151–162. 10322469 10.1016/s1364-6613(99)01299-1

[25] Varela F, Lachaux JP, Rodriguez E, Martinerie J (2001) The brainweb: phase synchronization and large-scale integration. Nat Rev Neurosci 2: 229–239. doi: 10.1038/35067550 11283746 10.1038/35067550

[26] von Stein A, Sarnthein J (2000) Different frequencies for different scales of cortical integration: from local gamma to long range alpha/theta synchronization. Int J Psychophysiol 38: 301–313. 11102669 10.1016/s0167-8760(00)00172-0

[27] Counsell C, Dennis M (2001) Systematic review of prognostic models in patients with acute stroke. Cerebrovasc Dis 12: 159–170. 11641579 10.1159/000047699

[28] Chen CC, Kiebel SJ, Friston KJ (2008) Dynamic causal modelling of induced responses. Neuroimage 41: 1293–1312. doi: 10.1016/j.neuroimage.2008.03.026 18485744 10.1016/j.neuroimage.2008.03.026

[29] Friston KJ, Harrison L, Penny W (2003) Dynamic causal modelling. Neuroimage 19: 1273–1302. 12948688 10.1016/s1053-8119(03)00202-7

[30] Fugl-Meyer AR, Jaasko L, Leyman I, Olsson S, Steglind S (1975) The post-stroke hemiplegic patient. 1. a method for evaluation of physical performance. Scand J Rehabil Med 7: 13–31. 1135616

[31] Mathiowetz V, Volland G, Kashman N, Weber K (1985) Adult norms for the Box and Block Test of manual dexterity. Am J Occup Ther 39: 386–391. 3160243 10.5014/ajot.39.6.386

[32] Wolf SL, Catlin PA, Ellis M, Archer AL, Morgan B, Piacentino A (2001) Assessing Wolf motor function test as outcome measure for research in patients after stroke. Stroke 32: 1635–1639. 11441212 10.1161/01.str.32.7.1635

[33] Wahl AS, Schwab ME (2014) Finding an optimal rehabilitation paradigm after stroke: enhancing fiber growth and training of the brain at the right moment. Front Hum Neurosci 8: 381 doi: 10.3389/fnhum.2014.00381 25018717 10.3389/fnhum.2014.00381 PMC4072965

[34] Kotter R (2004) Online retrieval, processing, and visualization of primate connectivity data from the CoCoMac database. Neuroinformatics 2: 127–144. doi: 10.1385/NI:2:2:127 15319511 10.1385/NI:2:2:127

[35] Stephan KE, Kamper L, Bozkurt A, Burns GA, Young MP, Kotter R (2001) Advanced database methodology for the Collation of Connectivity data on the Macaque brain (CoCoMac). Philos Trans R Soc Lond B Biol Sci 356: 1159–1186. doi: 10.1098/rstb.2001.0908 11545697 10.1098/rstb.2001.0908 PMC1088509

[36] Grefkes C, Nowak DA, Eickhoff SB, Dafotakis M, Kust J, Karbe H, et al (2008) Cortical connectivity after subcortical stroke assessed with functional magnetic resonance imaging. Ann Neurol 63: 236–246. doi: 10.1002/ana.21228 17896791 10.1002/ana.21228

[37] Herz DM, Christensen MS, Reck C, Florin E, Barbe MT, Stahlhut C, et al (2012) Task-specific modulation of effective connectivity during two simple unimanual motor tasks: a 122-channel EEG study. Neuroimage 59: 3187–3193. doi: 10.1016/j.neuroimage.2011.11.042 22146753 10.1016/j.neuroimage.2011.11.042

[38] Bonstrup M, Schulz R, Feldheim J, Hummel FC, Gerloff C (2016) Dynamic causal modelling of EEG and fMRI to characterize network architectures in a simple motor task. Neuroimage 124: 498–508. doi: 10.1016/j.neuroimage.2015.08.052 26334836 10.1016/j.neuroimage.2015.08.052

[39] Penny WD, Stephan KE, Mechelli A, Friston KJ (2004) Comparing dynamic causal models. Neuroimage 22: 1157–1172. doi: 10.1016/j.neuroimage.2004.03.026 15219588 10.1016/j.neuroimage.2004.03.026

[40] Stephan KE, Penny WD, Daunizeau J, Moran RJ, Friston KJ (2009) Bayesian model selection for group studies. Neuroimage 46: 1004–1017. doi: 10.1016/j.neuroimage.2009.03.025 19306932 10.1016/j.neuroimage.2009.03.025 PMC2703732

[41] Stephan KE, Penny WD, Moran RJ, den Ouden HE, Daunizeau J, Friston KJ (2010) Ten simple rules for dynamic causal modeling. Neuroimage 49: 3099–3109. doi: 10.1016/j.neuroimage.2009.11.015 19914382 10.1016/j.neuroimage.2009.11.015 PMC2825373

[42] Talelli P, Wallace A, Dileone M, Hoad D, Cheeran B, Oliver R, et al (2012) Theta burst stimulation in the rehabilitation of the upper limb: a semirandomized, placebo-controlled trial in chronic stroke patients. Neurorehabil Neural Repair 26: 976–987. doi: 10.1177/1545968312437940 22412171 10.1177/1545968312437940 PMC3719964

[43] Guyon I, Elisseeff A (2003) An introduction to variable and feature selection. Journal of machine learning research 3(mar): 1157–1182.

[44] Saeys Y, Inza I, Larranaga P (2007) A review of feature selection techniques in bioinformatics. Bioinformatics 23: 2507–2517. doi: 10.1093/bioinformatics/btm344 17720704 10.1093/bioinformatics/btm344

[45] Pandian S, Arya KN (2014) Stroke-related motor outcome measures: do they quantify the neurophysiological aspects of upper extremity recovery? J Bodyw Mov Ther 18: 412–423. doi: 10.1016/j.jbmt.2013.11.006 25042312 10.1016/j.jbmt.2013.11.006

[46] Bushnell C, Bettger JP, Cockroft KM, Cramer SC, Edelen MO, Hanley D, et al (2015) Chronic Stroke Outcome Measures for Motor Function Intervention Trials: Expert Panel Recommendations. Circ Cardiovasc Qual Outcomes 8: S163–169. doi: 10.1161/CIRCOUTCOMES.115.002098 26515205 10.1161/CIRCOUTCOMES.115.002098 PMC5289112

[47] Pandian S, Arya KN, Kumar D (2016) Minimal clinically important difference of the lower-extremity fugl-meyer assessment in chronic-stroke. Top Stroke Rehabil 23: 233–239. doi: 10.1179/1945511915Y.0000000003 27086865 10.1179/1945511915Y.0000000003

[48] Lang CE, Edwards DF, Birkenmeier RL, Dromerick AW (2008) Estimating minimal clinically important differences of upper-extremity measures early after stroke. Arch Phys Med Rehabil 89: 1693–1700. doi: 10.1016/j.apmr.2008.02.022 18760153 10.1016/j.apmr.2008.02.022 PMC2819021

[49] Finnigan SP, Walsh M, Rose SE, Chalk JB (2007) Quantitative EEG indices of sub-acute ischaemic stroke correlate with clinical outcomes. Clin Neurophysiol 118: 2525–2532. doi: 10.1016/j.clinph.2007.07.021 17889600 10.1016/j.clinph.2007.07.021

[50] Cramer SC, Nelles G, Benson RR, Kaplan JD, Parker RA, Kwong KK, et al (1997) A functional MRI study of subjects recovered from hemiparetic stroke. Stroke 28: 2518–2527. 9412643 10.1161/01.str.28.12.2518

[51] Caramia MD, Palmieri MG, Giacomini P, Iani C, Dally L, Silvestrini M (2000) Ipsilateral activation of the unaffected motor cortex in patients with hemiparetic stroke. Clin Neurophysiol 111: 1990–1996. 11068234 10.1016/s1388-2457(00)00430-2

[52] Cuadrado ML, Egido JA, Gonzalez-Gutierrez JL, Varela-De-Seijas E (1999) Bihemispheric contribution to motor recovery after stroke: A longitudinal study with transcranial doppler ultrasonography. Cerebrovasc Dis 9: 337–344. 10545692 10.1159/000016009

[53] Jones RD, Donaldson IM, Parkin PJ (1989) Impairment and recovery of ipsilateral sensory-motor function following unilateral cerebral infarction. Brain 112 (Pt 1): 113–132. 2917274 10.1093/brain/112.1.113

[54] Ward NS (2005) Mechanisms underlying recovery of motor function after stroke. Postgrad Med J 81: 510–514. doi: 10.1136/pgmj.2004.030809 16085742 10.1136/pgmj.2004.030809 PMC1743338

[55] Johansen-Berg H, Rushworth MF, Bogdanovic MD, Kischka U, Wimalaratna S, Matthews PM (2002) The role of ipsilateral premotor cortex in hand movement after stroke. Proc Natl Acad Sci U S A 99: 14518–14523. doi: 10.1073/pnas.222536799 12376621 10.1073/pnas.222536799 PMC137915

[56] Furl N, Coppola R, Averbeck BB, Weinberger DR (2014) Cross-frequency power coupling between hierarchically organized face-selective areas. Cereb Cortex 24: 2409–2420. doi: 10.1093/cercor/bht097 23588186 10.1093/cercor/bht097 PMC4128705

[57] Hayashi K, Sawa T, Matsuura M (2008) Anesthesia depth-dependent features of electroencephalographic bicoherence spectrum during sevoflurane anesthesia. Anesthesiology 108: 841–850. doi: 10.1097/ALN.0b013e31816bbd9b 18431119 10.1097/ALN.0b013e31816bbd9b

[58] Hagihira S, Takashina M, Mori T, Mashimo T, Yoshiya I (2002) Changes of electroencephalographic bicoherence during isoflurane anesthesia combined with epidural anesthesia. Anesthesiology 97: 1409–1415. 12459666 10.1097/00000542-200212000-00012

[59] Nunez PL, Srinivasan R, Fields RD (2015) EEG functional connectivity, axon delays and white matter disease. Clin Neurophysiol 126: 110–120. doi: 10.1016/j.clinph.2014.04.003 24815984 10.1016/j.clinph.2014.04.003 PMC5018992

[60] Finnigan SP, Rose SE, Chalk JB (2008) Contralateral hemisphere delta EEG in acute stroke precedes worsening of symptoms and death. Clin Neurophysiol 119: 1690–1694. doi: 10.1016/j.clinph.2008.03.006 18450505 10.1016/j.clinph.2008.03.006

[61] Kuypers HGJM, Martin GF (1982) Anatomy of descending pathways to the spinal cord. Amsterdam; New York New York: Elsevier Biomedical Press; Sole distributors for the USA and Canada, Elsevier Science Pub x, 411 p. p.

