# Supplementary figures and images for: EEG-based motor network biomarkers for identifying target patients with stroke for upper limb rehabilitation and its construct validity
Source: PLoS One. 2017 Jun 14;12(6):e0178822. doi: 10.1371/journal.pone.0178822 (PMC5470671; doi:10.1371/journal.pone.0178822)

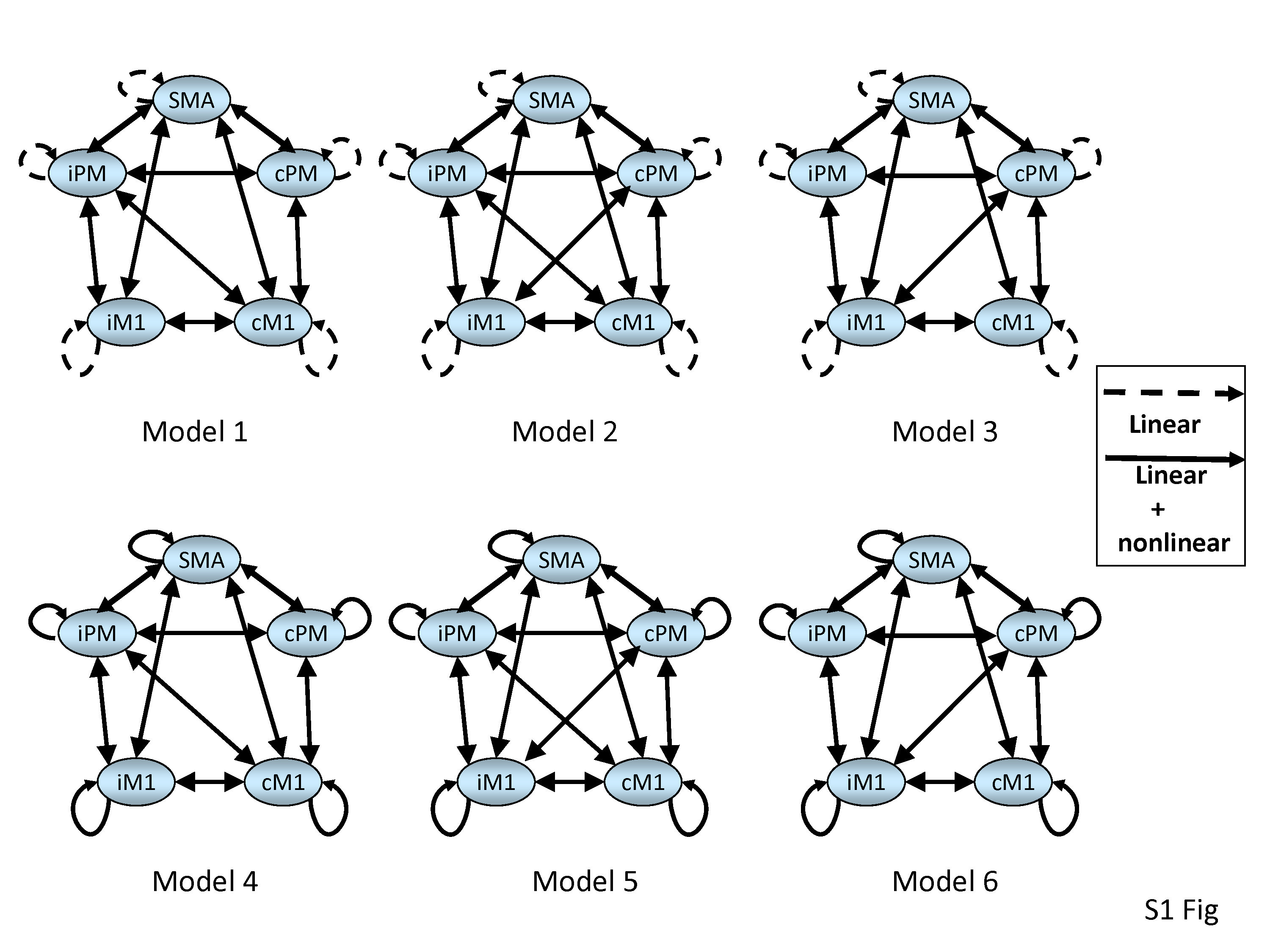

Supplement: S1 Fig — i: ipsilesional; c: contralesional; M1: primary motor cortex; PM: premotor cortex; SMA: supplementary motor area. (TIF) [file pone.0178822.s001.tif]
